# A DNA Vaccine Incorporating the MHC Class I Trafficking Domain and PADRE Epitope Enhances Antitumor Immunity in a Murine Pancreatic Cancer Model

**DOI:** 10.3390/ijms27042039

**Published:** 2026-02-22

**Authors:** Simiao Cao, Guoxuan Bai, Qimuge Wuri, Jiayin Li, Xiaojing Zhang, Zhilin Han, Hui Wu, Jiaxin Wu, Chu Wang, Xianghui Yu, Haihong Zhang

**Affiliations:** 1 National Engineering Laboratory for AIDS Vaccine, School of Life Science, Jilin University, Changchun 130012, China; 17865620671@163.com (S.C.); baigx1322@mails.jlu.edu.cn (G.B.); wrqmg24@mails.jlu.edu.cn (Q.W.); lijiayin220204@163.com (J.L.); zhangxj25@jlu.edu.cn (X.Z.); zhlhan123@163.com (Z.H.); topwuhui@jlu.edu.cn (H.W.); wujiaxin@jlu.edu.cn (J.W.); wangchu18@jlu.edu.cn (C.W.); xianghui@jlu.edu.cn (X.Y.); 2 Key Laboratory for Molecular Enzymology and Engineering, The Ministry of Education, School of Life Sciences, Jilin University, Changchun 130012, China

**Keywords:** immunotherapy, DNA vaccines, MITD, PADRE, cross-presentation

## Abstract

DNA-based cancer vaccines represent a safe and promising immunotherapeutic strategy, but their clinical efficacy is often limited by weak immunogenicity, primarily due to inefficient antigen cross-presentation. To overcome this challenge, the MHC class I trafficking domain (MITD) can be fused to tumor antigens to enhance their intracellular routing in dendritic cells (DCs), thereby promoting the efficiency of cross-presentation. In addition, incorporation of CD4^+^ T cell epitopes, such as PADRE or P2P16, can robustly activate CD4^+^ T cells, further amplifying antitumor immunity. Thus, combining MITD with CD4^+^ epitopes is expected to synergistically improve DNA vaccine potency. Mesothelin (MSLN), a tumor-associated antigen highly expressed in pancreatic cancer, was selected as the target in this study. We designed MSLN-targeted DNA vaccines incorporating MITD together with either PADRE or P2P16. In a Panc02 murine model, the MITD–PADRE construct, a novel design, elicited stronger immune responses and more effective antitumor activity compared to other formulations. To further counteract immunosuppression, we combined the vaccine with gemcitabine, which enhanced therapeutic efficacy. Together, these findings demonstrate that integrating PADRE with MITD in MSLN-targeted DNA vaccines offers a promising combinatorial strategy for advancing pancreatic cancer immunotherapy.

## 1. Introduction

Pancreatic cancer ranks among the most lethal malignancies, accounting for 4.8% of cancer-related deaths, underscoring an urgent need for novel therapeutic strategies [1,2]. While cancer immunotherapy has made extraordinary strides in recent years, DNA tumor vaccines are particularly noteworthy due to their ability to activate CD8^+^ T cell immunity, high safety profile, and ease of production and storage [3]. However, obstacles such as poor immunogenicity have hindered the clinical translation of DNA vaccines. An effective DNA cancer vaccine must induce robust and sustained T cell responses, which critically depend on efficient antigen processing and presentation via MHC class I molecules. Notably, antigen cross-presentation by MHC class I molecules is an exceptionally inefficient process, with only ~150 peptides presented per second out of approximately 2 million generated [4,5,6,7].

The MHC class I trafficking domain (MITD), located within the cytoplasmic domain of the MHC class I molecule, contains a tyrosine-based sorting signal responsible for its recycling between endolysosomal compartments [8]. Cross-presentation of exogenous antigens by major histocompatibility complex (MHC) class I molecules is primarily mediated through two distinct pathways: the vacuolar pathway and the cytosolic pathway. In the vacuolar pathway, exogenous antigens are internalized via endocytosis and trafficked through endosomal compartments to lysosomes, where they are proteolytically degraded into antigenic peptides. Concurrently, MHC class I molecules are recycled from the plasma membrane or directly delivered from the Golgi apparatus to late endosomes, where the acidic microenvironment facilitates peptide exchange and loading. In the cytosolic pathway, internalized antigens are translocated from endosomal compartments into the cytosol and degraded by the proteasome. The shortened peptides are subsequently transported into the endoplasmic reticulum (ER) via TAP for loading onto MHC class I molecules, followed by trafficking to the cell surface. Alternatively, proteasome-generated peptides may be reimported into endosomal compartments, where they are loaded onto MHC class I molecules prior to surface presentation. The MITD mediates the targeting of MHC class I molecules to endosomal compartments through its tyrosine-based sorting motif (YXXA), thereby facilitating the loading of exogenous peptides and enhancing antigen cross-presentation [7]. Recent studies indicate that antigens conjugated to MITD exploit this dynamic trafficking pathway. They are rerouted to specific cellular compartments associated with antigen processing and presentation—including the endoplasmic reticulum (ER), Golgi apparatus, plasma membrane, lysosomes, and endosomes—depending on the maturation stage of dendritic cells (DCs) [6,9,10]. This precise intracellular compartmentalization consequently facilitates efficient antigen cross-presentation [8,11].

Incorporation of T-cell epitope peptides into cancer vaccines has been shown to promote immune cell activation, thereby enhancing vaccine immunogenicity [12]. Consequently, incorporating a CD4^+^ T cell epitope into the MITD construct represents a potential strategy to further enhance CD4^+^ T cell activation. The universal CD4^+^ T cell epitope P2P16, derived from tetanus toxin, has been widely used in clinical applications. For example, the first clinical-stage tumor vaccine BNT116 employed a P2P16-MITD sequence as combined adjuvant elements [13,14,15]. Alternatively, the pan-DR epitope (PADRE), a synthetic peptide engineered to bind diverse HLA-DR alleles, has been shown to elicit more potent CD4^+^ T cell responses than P2P16 [16,17]. Therefore, attaching PADRE to the MITD sequence offers a novel strategy to enhance cellular immune responses induced by DNA vaccines targeting pancreatic cancer.

The selection of an appropriate antigen is critical for the efficacy of DNA tumor vaccines [18]. Mesothelin (MSLN), a GPI-anchored cell surface glycoprotein, is synthesized as a 69 kDa precursor protein and subsequently cleaved into two products: megakaryocyte potentiating factor (MPF) and mature membrane-bound MSLN [19,20,21,22]. Many MSLN-targeted therapeutic approaches for pancreatic cancer have been developed, including MSLN-directed antibodies, CAR-T cells, and vaccines. As a tumor-associated antigen, MSLN is highly expressed in pancreatic tumors but exhibits minimal expression in normal human tissues [23,24]. It is also reported to promote tumor cell invasion and migration by inducing epithelial–mesenchymal transition (EMT), significantly worsening pancreatic cancer prognosis [18,25,26]. While MSLN exhibits moderate intrinsic immunogenicity, its tumor-restricted expression and functional role in malignancy collectively make it an ideal target for pancreatic cancer immunotherapy.

Dendritic cells (DCs) uptake, process, and present tumor antigens via MHC class I or II molecules, with cross-presentation being a critical pathway for MHC class I loading [27]. As type 1 conventional dendritic cells (cDC1s) are the primary mediators of MHC class I cross-presentation, our previous research built upon cDC1-targeting strategy via a vaccine platform based on XCL1 fusion [28]. In this study, we employed two distinct chimeric elements—P2P16-MITD and PADRE-MITD—to enhance both CD4^+^ helper T cell activation and cross-presentation to promote immunogenicity of MSLN-targeted DNA vaccines. Furthermore, to reduce immunosuppression within the tumor microenvironment, we combined DNA vaccine with gemcitabine, aiming to boosting their antitumor efficacy [29,30]. Our findings demonstrate that this combination therapy significantly enhances antitumor responses in a murine pancreatic cancer model, establishing a promising translational approach for pancreatic cancer treatment.

## 2. Results

### 2.1. The Construction and Validation of DNA Vaccines

**Figure 1:**
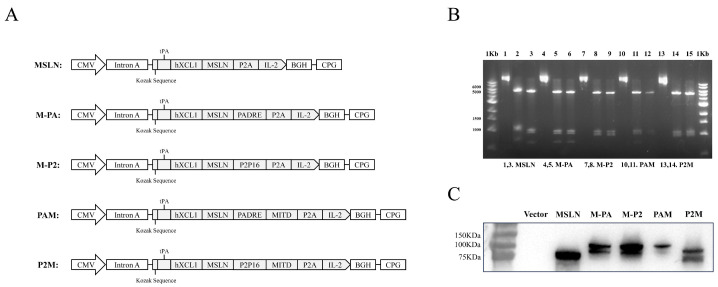
Construction and validation of DNA tumor vaccines. (**A**) Schematic diagrams of the DNA plasmids used in this study. (**B**) The DNA plasmids were digested with Pst I/BamH I and were identified by Agarose gel electrophoresis. (**C**) 293T cells were transfected with the DNA plasmids, and the expression of the fusion proteins was verified by Western blotting, using an anti-MSLN antibody as the primary antibody and HRP-conjugated Goat IgG as the secondary antibody.

Mesothelin is a tumor-associated antigen which is highly expressed in pancreatic tumor cells. Leveraging on our previously established vaccine platform [28], we initially constructed a MSLN-targeted DNA vaccine. The DNA vaccine encodes a fusion protein comprising human XCL1(hXCL1) to target cDC1, MSLN as the primary antigen, and P2A peptide-linked IL-2 as a molecular adjuvant. Subsequently, we constructed two additional vaccines: CpVR-hXCL1/MSLN/PADRE-IL-2 (M-PA) and CpVR-hXCL1/MSLN/P2P16-IL-2 (M-P2) to evaluate the additive effects of the two CD4^+^ T cell epitopes PADRE/P2P16 on the DNA vaccines. Furthermore, to enhance the efficiency of antigen cross-presentation, we constructed two optimized DNA vaccines PAM/P2M by incorporating the MITD to the end of the PADRE/P2P16 gene (Figure 1A). The plasmid was verified by double digestion with Pst I/BamH I (Figure 1B), and the expression of the fusion proteins was analyzed by Westen blotting using an anti-MSLN antibody (Figure 1C).

### 2.2. The Incorporation of MITD Significantly Enhances the Immunogenicity of DNA Vaccines

**Figure 2:**
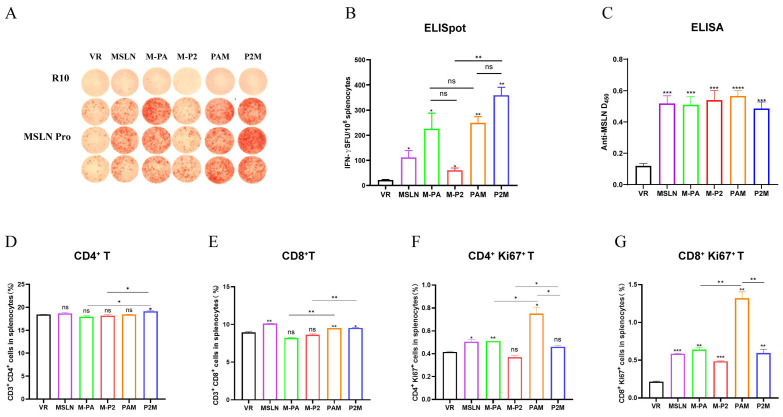
Immunogenicity analysis of DNA vaccines. (**A**,**B**) Representative images of ELISpot and quantification of IFN-γ FSC in different immune groups (*n* = 5) after stimulation. MSLN Pro was utilized as specific stimulator. (**C**) Levels of MSLN-specific binding antibodies in the sera of mice were measured by ELISA. (**D**–**G**) The proportion of CD4^+^ T cells activation (**D**), CD8^+^ T cells activation (**E**), proliferative CD4^+^ T cells (**F**), proliferative CD8^+^ T cells (CD8^+^Ki67^+^) (**G**) in splenocytes were measured by flow cytometry. * *p* < 0.05, ** *p* < 0.01, *** *p* < 0.001, **** *p* < 0.0001, and ns: no significance. Except where specifically noted, all significant analyses are compared with the VR group.

To evaluate the systemic immunogenicity of the DNA vaccines in vivo, C57BL/6 mice were immunized with 100 μg DNA vaccines by fast DNA immunization strategy [31,32] on day 0, 2, and 5 to compare the immunogenicity of the vaccines in vivo. Antigen-specific T cell responses were subsequently quantified via an IFN-γ ELISpot assay. Upon stimulation with MSLN protein, all five candidates successfully primed MSLN-specific T cells to secrete IFN-γ. These responses were significantly elevated compared to the VR control group, thereby confirming the robust intrinsic immunogenicity of the MSLN antigen within this platform. Notably, the M-PA construct elicited a higher frequency of IFN-γ-secreting cells than M-P2; however, the incorporation of MITD sequence reversed this trend, with the P2M group exhibiting superior spot-forming units compared to the PAM group (Figure 2A,B). Parallel assessments of humoral immunity via ELISA revealed that all vaccinated mice developed significantly higher anti-MLSN antibody titers than the control group (Figure 2C). While the PAM group achieved the highest absolute antibody levels, the increment did not reach statistical significance across the vaccine cohorts. Delving deeper into cellular dynamics, we observed that the individual integration of P2P16 or PADRE (M-P2 and M-PA) failed to significantly boost the overall frequency of CD4^+^ and CD8^+^ T cells relative to the VR group (Figure 2D,E). Nevertheless, the PAM group displayed a distinct advantage in T cell proliferative capacity. Crucially, the addition of the MITD sequence functioned as a potent enhancer, markedly augmenting CD4^+^ and CD8^+^ T cell proliferation in the PAM group and underscoring its role in potentiating cellular immune effector functions (Figure 2F,G). Given these divergent immunological profiles, both PAM and P2M were prioritized for subsequent challenge experiments to determine their definitive antitumor efficacy.

### 2.3. PADRE-MITD Exhibits Superior Antitumor Efficacy Compared with P2P16-MITD in a Murine Pancreatic Cancer Model

**Figure 3:**
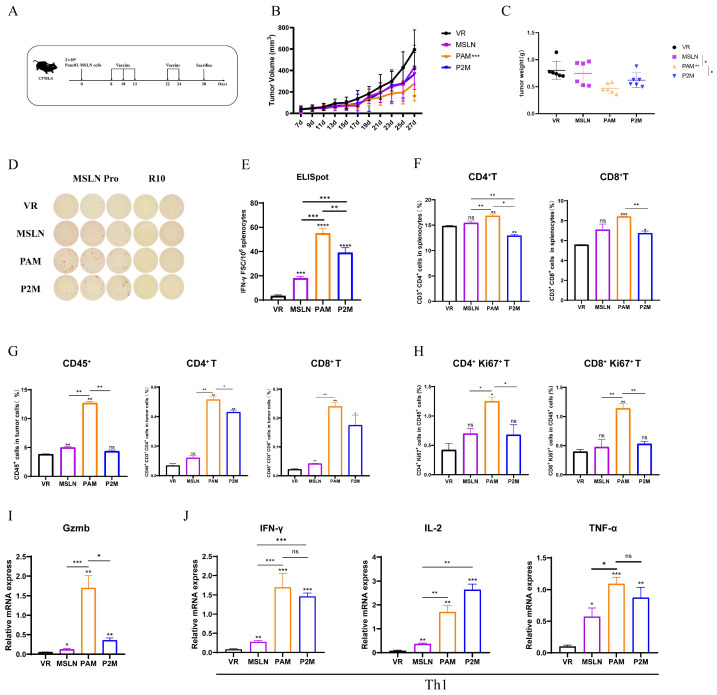
Antitumor effect of the DNA vaccines and detection of related cellular immune responses. (**A**) Schematic diagram of the DNA vaccination strategy. C57BL/6 mice (*n* = 6) were inoculated with 2 × 10^6^ MSLN-EGFP^+^ Panc02 cells at day 0 and received treatment on day 8, 10, 13, 22 and day 24. The mice were euthanized on day 30 after tumor challenge. (**B**) Tumor growth curve was measured every two days. (**C**) Tumor weight analysis in different immunized groups. (**D**,**E**) Representative images of ELISpot and quantification of IFN-γ FSC in different immune groups after stimulation. MSLN Pro was utilized as specific stimulator. (**F**) The Proportion of CD3^+^CD4^+^ T cells and CD3^+^CD8^+^ T cells in splenocytes. (**G**,**H**) Proportion of tumor infiltrating lymphocytes, CD45^+^CD3^+^CD4^+^ T cells, CD45^+^CD3^+^CD8^+^ T cells (**G**) and proliferative CD4^+^ T cells (CD4^+^Ki67^+^), proliferative CD8^+^ T cells (CD8^+^Ki67^+^) (**H**) were measured by flow cytometry. The expression levels of Gzmb (**I**) and Th1 cytokine (IFN-γ, IL-2, TNF-α) (**J**) were measured by qRT-PCR. * *p* < 0.05, ** *p* < 0.01, *** *p* < 0.001, **** *p* < 0.0001, and ns: no significance. Except where specifically noted, all significant analyses are compared with the VR group.

Next, we evaluated the therapeutic efficacy of MITD-incorporated DNA vaccines using a pancreatic cancer mouse model stably expressing MSLN (MSLN-EGFP^+^ Panc02, Figure S1A–C). C57BL/6 mice (*n* = 6) were inoculated with 2 × 10^6^ MSLN-EGFP^+^ Panc02 cells on day 0 and received treatment on day 8. Based on the fast DNA immunization strategy described above, two additional immunizations were administered on day 9 (day 22) and day 11 (day 24) with a 100 μg vaccine dose each time (Figure 3A). Our previous research demonstrated that this regimen achieves better antitumor immune responses. Compared to the VR control, tumor growth was markedly suppressed in mice treated with MSLN, PAM or P2M (Figure 3B,C). Furthermore, all three therapeutic cohorts exhibited significant reductions in both tumor volume and weight, with the PAM group demonstrating the most pronounced inhibitory effect.

To elucidate the underlying immune mechanisms, we first quantified the systemic response via an IFN-γ ELISpot assay. All three vaccine groups (MSLN, PAM, P2M) mounted robust cellular immune responses (Figure 3D,E). Notably, PAM induced the most robust expansion of the MSLN-specific T cell pool. Analysis of the spot-forming units (SFUs) revealed that PAM elicited the highest frequency of individual effector T cells capable of secreting IFN-γ upon antigen re-encounter, representing a significant increase over both the MSLN and P2M groups. Then, we performed a flow cytometric analysis of immune cell populations in the spleen and tumor. In the spleen, the proportion of CD4^+^ T cells and CD8^+^ T cells in both PAM and P2M elicited a significant increase compared to the VR group (Figure 3F). Similarly, the most significant enrichment and proliferation of CD4^+^ and CD8^+^ T cells within the tumor-infiltrating lymphocytes were observed in the PAM group (Figure 3G,H and Figure S2). Finally, we analyzed the expression of Granzyme B (GzmB) and Th1-type cytokines (IFN-γ, IL-2 and TNF-α) in tumor tissues using qRT-PCR (Figure 3I,J). All therapeutic groups showed significant transcriptional upregulation of these markers. Specifically, the PAM group exhibited the highest levels of GzmB, suggesting enhanced cytotoxic activity by CD8^+^ T cells. Additionally, the superior expression of IFN-γ and TNF-α in the PAM group reflects its capacity to induce a potent and localized cellular immune milieu, which likely drives its superior antitumor efficacy.

### 2.4. PAM Induces Relatively Weaker Immunosuppression in Tumor Microenvironment

**Figure 4:**
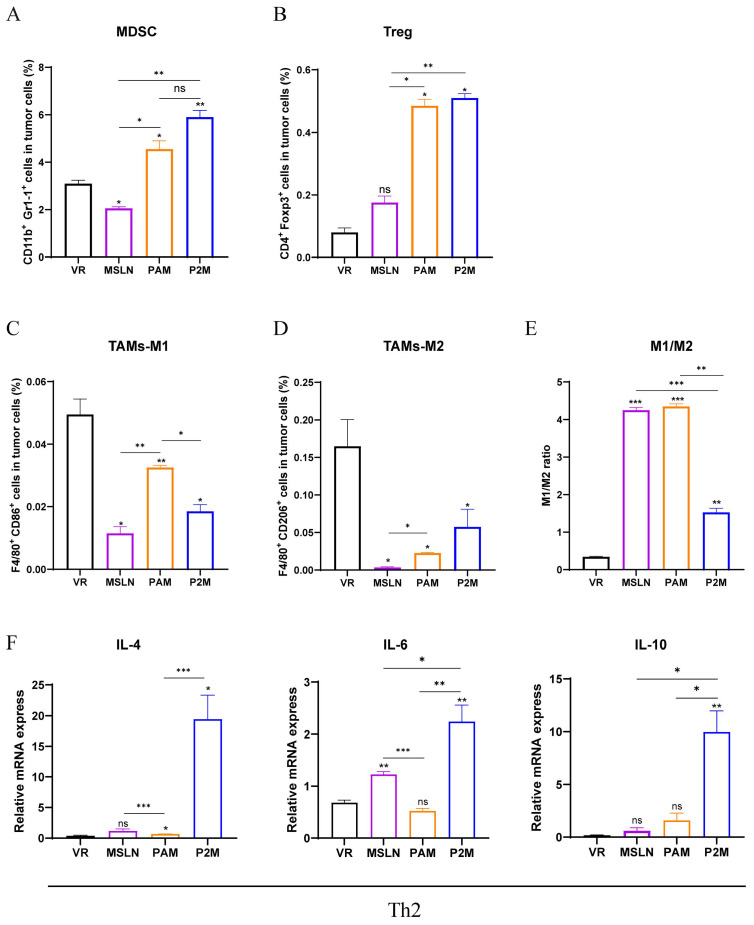
Analysis of intratumoral immunosuppression in immunized tumor-bearing mice. (**A**–**D**) The proportion of tumor-infiltrated MDSCs (**A**), Tregs (**B**), TAMs-M1 (**C**), TAMs-M2 (**D**) were measured by flow cytometry. (**E**) The ratio of M1/M2. (**F**) The expression levels of Th2 cytokines (IL-4, IL-6, IL-10) were measured by qRT-PCR. * *p* < 0.05, ** *p* < 0.01, *** *p* < 0.001, and ns: no significance. Except where specifically noted, all significant analyses are compared with the VR group.

To probe the potential mechanisms that hindered the antitumor effects of the vaccines, we shifted our focus to the tumor-infiltrating immunosuppressive populations and the underlying cytokine milieu. Using flow cytometric analysis, we identified a conspicuous recruitment of myeloid-derived suppressor cells (MDSCs) and regulatory T cells (Tregs) within the tumor tissue across all vaccinated groups (Figure 4A,B). While the P2M group presented with slightly higher frequencies of these inhibitory subsets compared to PAM, the variance remained statistically non-significant. The remodeling of tumor-associated macrophages (TAMs) was further examined to evaluate the inflammatory state of the microenvironment. Strikingly, the PAM treatment reprogrammed the macrophage landscape, yielding a significantly higher proportion of pro-inflammatory M1-type TAMs compared to other groups (Figure 4C). In contrast, we observed a significant decline in the immunosuppressive M2-type TAM population following vaccination in all therapeutic groups, with the PAM group exhibiting the most pronounced reduction (Figure 4D). This resulted in a superior M1/M2 polarization ratio in the PAM group, which was significantly relative to the P2M group, signifying a more favorable pro-immunogenic shift. (Figure 4E).

Regarding the transcriptional profile of the TME, qRT-PCR results indicated that Th2-type markers, specifically IL-4, IL-6, and IL-10, were upregulated post-immunization, particularly within the P2M group. (Figure 4F). As these cytokines are notorious for blunting antitumor immunity and fostering Treg stability, their enrichment aligns with the observed cellular patterns of suppression. Collectively, these findings underscore a dual-edged effect: while both vaccines activate effector responses, the PAM platform strikes a more advantageous balance by fostering a pro-inflammatory macrophage bias while minimizing the compensatory induction of Th2-mediated suppression. This highlights that synergizing the PAM vaccine with strategies to alleviate TME-mediated immunosuppression represents a promising approach to further bolster antitumor immunity.

### 2.5. Combination Therapy with Gem Elevated the Antitumor Efficacy of PAM

**Figure 5:**
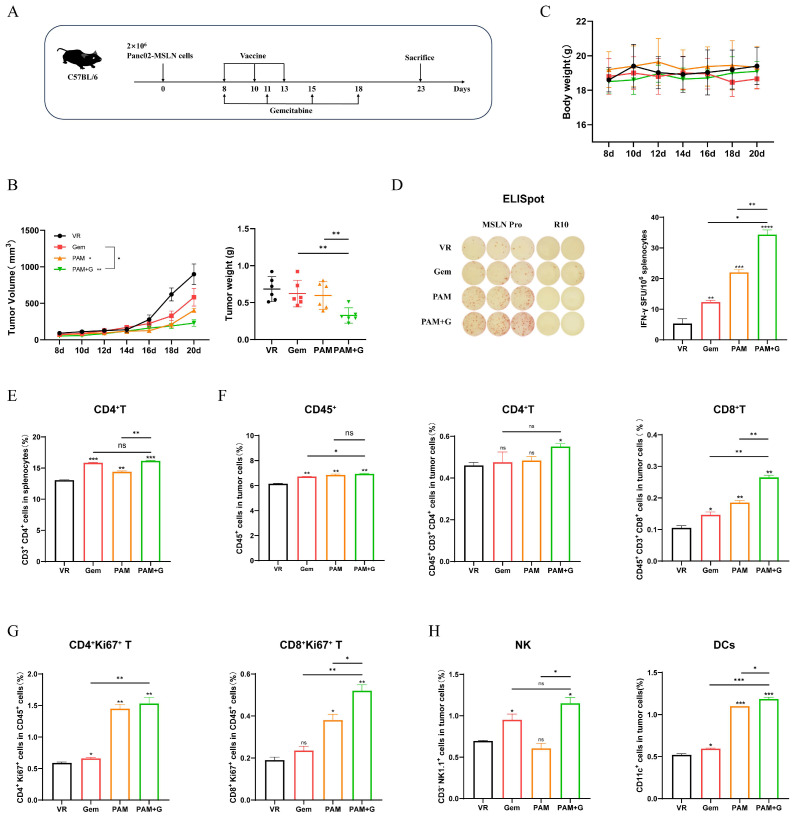
PAM combined with Gem further enhances antitumor effect. (**A**) Schematic diagram of DNA vaccine combined with Gem immunization strategy. C57BL/6 mice (*n* = 6) were inoculated with 2 × 10^6^ MSLN-EGFP^+^ Panc02 cells at day 0 and received DNA vaccine treatment on day 8, 10, 13, and Gem treatment on day 8, 11, 15, 18. (**B**) Tumor growth and weight were measured in different immunized groups. (**C**) Body weight change curves were measured every two days (Black: VR; Red: Gem; Orange: PAM; and Green: PAM+G). (**D**) Representative images of ELISpot and quantification of IFN-γ FSC in different immune groups after stimulation. MSLN Pro was utilized as specific stimulator. (**E**–**G**) The proportion of CD3^+^CD4^+^ T cells in splenocytes (**E**), the proportion of tumor infiltrating lymphocytes, CD45^+^CD3^+^CD4^+^ T cells, CD45^+^CD3^+^CD8^+^ T cells (**F**) and proliferative CD4^+^ T cells (CD4^+^Ki67^+^), proliferative CD8^+^ T cells (CD8^+^Ki67^+^) (**G**) were measured by flow cytometry. (**H**) The proportion of tumor-infiltrated NKs and DCs were measured by flow cytometry. * *p* < 0.05, ** *p* < 0.01, *** *p* < 0.001, **** *p* < 0.0001, and ns: no significance. Except where specifically noted, all significant analyses are compared with the VR group.

To further mitigate the immunosuppressive environment of the DNA vaccines, we combined gemcitabine (Gem) with the PAM vaccine, which proved the most efficient treatment regimen to test the therapeutic effect in the pancreatic cancer mouse model [33,34]. C57BL/6 mice were inoculated 2 × 10^6^ MSLN-EGFP^+^ Panc02 cells and started treatment on day 8 after tumor challenge. The mice received three injections of 100 μg of DNA vaccines on day 8, day 10 and day 13, along with intraperitoneal administration of Gem on day 8, day 11, day 15 and day 18 with a dose of 15 mg/kg (Figure 5A). Upon harvesting tumor tissues on day 23, assessments of tumor burden revealed that both monotherapies and the synergistic combination significantly attenuated tumor growth (Figure 5B). In particular, mice treated with the combination of PAM and Gem exhibited a profound reduction in both tumor volume and weight compared to the VR control or respective monotherapy groups. Furthermore, the safety profile was favorable, as body weights remained stable throughout the study. (Figure 5C). No obvious differences in general health were observed in the Gem-treated group, suggesting no distinct toxicity at the given dose.

ELISpot assay confirmed that the combination therapy induced significantly higher IFN-γ secretion compared to either PAM or Gem monotherapy, reflecting the synergistic effect between the two agents and the efficacy of the combination therapy (Figure 5D). Moreover, analysis of the systemic immune response showed a significant expansion of the CD4^+^ T cell population within the splenocytes of the combination group (Figure 5E); conversely, CD8^+^ T cell counts in the spleen remained comparable across all therapeutic groups.

Intratumoral analysis further corroborated these findings, as the combination group demonstrated a significant enrichment of CD45^+^ lymphocytes, CD4^+^ T cells, and CD8^+^ T cells (Figure 5F and Figure S3). These infiltration levels surpassed those observed in the monotherapy arms, with the proliferative capacity of CD4^+^ and CD8^+^ T cells peaking in the dual-therapy cohort (Figure 5G). Consistent with these results, the recruitment of innate effector cells, such as natural killer (NK) cells and dendritic cells (DCs), was also maximized under the combination regimen (Figure 5H), thereby reinforcing the superior immunostimulatory potential of this integrated therapeutic approach.

### 2.6. PAM Combined with Gemcitabine Further Reduces Immunosuppression in Tumor Microenvironment

**Figure 6:**
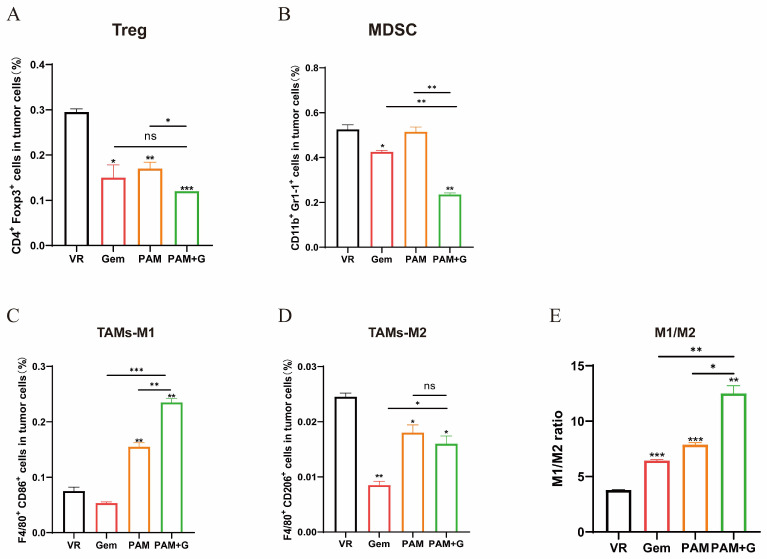
Impact of Gem combination therapy on intratumoral immunosuppression. (**A**–**D**) The proportion of tumor infiltrated MDSCs (**A**), Tregs (**B**), TAMs-M1 (**C**), TAMs-M2 (**D**) were measured by flow cytometry. (**E**) The ratio of M1/M2. * *p* < 0.05, ** *p* < 0.01, *** *p* < 0.001, and ns: no significance. Except where specifically noted, all significant analyses are compared with the VR group.

To elucidate the effect of combination therapy on the tumor immunosuppressive microenvironment, we analyzed the intratumoral infiltrated immunosuppressive cells. Both Tregs and MDSCs in the combination group indicated a significant decrease in contrast to either the VR group or the PAM vaccine monotherapy groups (Figure 6A,B). The combination group induced significantly higher levels of M1-TAMs and lower levels of M2-TAMs compared to the other groups, and the M1/M2 ratio indicated a pronounced shift toward a pro-immunogenic phenotype, which was the strongest among all the therapeutic groups and thus interprets a potential factor for the superior antitumor efficacy of the combination group as is presented in the tumor growth curve (Figure 6C–E).

## 3. Discussion

The efficacy of a DNA cancer vaccine is primarily influenced by factors such as appropriate antigen selection, efficient antigen presentation and induction of robust CD4^+^ T helper cells and Cytotoxic CD8^+^ T cells activation [18]. MSLN overexpression is observed in 80–85% of pancreatic cancer, while its expression in normal tissues is minimal [35,36]. Therefore, MSLN serves as an ideal target for pancreatic cancer immunotherapy, providing high tumor specificity and reducing the risk of off-target toxicity in normal tissues. However, due to the inherently limited immunogenicity of DNA vaccines, the vaccine containing MSLN antigen alone is insufficient to elicit a robust immune response capable of activating both CD4^+^ and CD8^+^ T cells for effective antitumor activity [37,38]. CD4^+^ T cells play a pivotal role in tumor immunity, which promotes the reprogramming of CD8^+^ T cells and facilitates the induction of both effector and memory CD8^+^ T cell responses within the tumor microenvironment. CD4^+^ T helper cells secrete IL-2 to recruit the CD8^+^ T cells, which promotes their proliferation and upregulates granzyme B expression for the killing of target tumor cells [39,40]. Thus, we incorporated two universal CD4^+^ T cell epitope candidates, P2P16 and PADRE, which are capable of binding to diverse HLA-II molecules and stimulate CD4^+^ T cell activation. We compared their individual effects on immune enhancement. Although the results indicated that PADRE contributed to stronger IFN-γ secretion than P2P16, simply incorporating a CD4^+^ T cell epitope was insufficient to profoundly enhance the immunogenicity through immune cell activation. Consequently, we focused on additional factors that could potentially affect the immunogenicity of the vaccines and further attached another adjuvant element MITD to the CD4^+^ T cell epitopes. As hypothesized, MITD-attached vaccines indeed emerged an enhanced capability of inducing antigen-specific immune responses, which is likely due to the intracellular trafficking function mediated by MITD in immature and mature DCs, boosting the efficacy of antigen cross-presentation. Intriguingly, P2P16 conjugated to MITD elicited a stronger IFN-γ secretion in the ELISpot assay compared to PADRE-MITD, which is not compatible with the results observed when the two epitopes were used individually. Reports suggest that antigens attached to MITC would be rerouted into compartments where peptides are derived from MHC I and loaded on MHC II molecules, which might be more beneficial for the processing of P2P16 within the endosomal or ER-associated pathways [7,41,42].

Due to the distinct immunogenic fluctuation in P2P16 and PADRE conjugated to MITD, both constructs, along with the MSLN group, were selected for the subsequential antitumor efficacy study. The results indicate that the PAM group exhibited remarkably superior antitumor effects and a higher level of in vivo immune response compared to the P2M group. We noticed that the declining intratumoral immunosuppressive cells in the PAM group may underlie the better antitumor efficacy compared to the P2M group. This highlights the potential of combination therapy which is conceived to mitigate the intratumoral immunosuppression. Several studies revealed that low-dose gemcitabine could promote to the depletion of regulatory T cells and reduce MDSCs production [43,44,45]. Therefore, we combined Gem with the PAM vaccine, and our results showed that the combination therapy significantly decreased intratumoral MDSCs and Tregs and inhibited tumor growth.

Notably, the reliance on the single Panc02 murine model is lacks validation in more complex systems with dense stroma and mutational diversity of human PDAC; however, this cold tumor model served as a rigorous platform for verifying the adjuvant scaffold PADRE-MITD in the ability of comprehensive immunostimulatory. While these results provide essential preliminary evidence, further validation in more complex systems such as KPC spontaneous models or PDX humanized mice, as well as multiple cell lines, will be the focus of our future research to better evaluate the vaccine’s clinical potential.

In conclusion, our finding first indicates that the incorporation of the adjuvant-elements regimen PADRE-MITD profoundly promoted the immunogenicity and antitumor effect of the MSLN-targeted vaccine through CD4^+^ helper T cell activation and cross-presentation enhancement. To mitigate the immunosuppression associated with the DNA vaccine, a gemcitabine combination therapy was employed and exhibited promising therapeutic efficacy. Our findings provide a novel strategy for pancreatic cancer treatment that overcomes the obstacles of previous MSLN-targeted DNA vaccines, such as the insufficiency in antigen cross-presentation and the immunosuppression induced within the tumor microenvironment, underscoring a critical approach in clinical trials.

## 4. Materials and Methods

### 4.1. Animals and Cell Lines

Female C57BL/6 mice (6–8 weeks old) were purchased from Liaoning Changsheng Biotechnology Co., Ltd. (Benxi, China) and raised in the animal experiment platform of the College of Life Sciences, Jilin University. Mice were randomly allocated to experimental groups for immunogenicity studies (*n* = 5) and antitumor efficacy assays (*n* = 6). All procedures were performed in accordance with Chinese law and were approved by the Ethics Committees of Jilin University. The murine Panc02 cell lines were provided by the National Center for Nanoscience and Technology.

The stable-expressing MSLN-EGFP^+^ Panc02 cell line was constructed in our lab. The pLVX-MSLN-EGFP lentiviral expression vector was constructed and co-transfected into 293T cells with 4 μg of pLVX-MSLN-EGFP, 5 μg of psPAX2 and 2.5 μg VSVG using Lipofactamine 2000 (Invitrogen, Carlsbad, CA, USA). Viral supernatants were collected and concentrated for subsequent use. Panc02 cells were seeded in 6-well plates and cultured at 37 °C in a CO_2_ incubator. Concentrated lentiviral particles were then added to each well along with 10 μL of polybrene to enhance infection. After 24 h, the medium was replaced with DMEM (Hyclone, South Logan, UT, USA) containing 10% FBS (Kangyuan Biology, Mingguang, China). Following an additional 48 h of incubation, puromycin (Absin, Shanghai, China) was added at a final concentration of 6 μg/mL for selection. After 7 days of selection, positively transduced cells were sorted by flow cytometry and expanded for further use.

### 4.2. Vaccine Construction

The empty vector VR1012 (VR) served as the placebo control, which is provided by and maintained in our laboratory. The plasmid pLVX-MSLN was purchased from Wuhan Miaoling Biotechnology Co., Ltd. (Wuhan, China). The plasmid CpVR-P2M and CpVR-PAM were synthesized by Nanjing Jinsirui Biotechnology Co., Ltd. (Nanjing, China). The plasmids MSLN, M-PA, M-P2, PAM, and P2M were constructed using the Seamless Assembly Cloning Kit (TransGen Biotech, Beijing, China) according to the manufacturer’s instructions. The inserts were cloned into the Pst I/BamH I sites of the VR1012 vector, using the previously constructed plasmid CpVR-XCL1-MUC1 from our laboratory as the template. All plasmids were validated through DNA sequencing and Western blot analysis was performed to determine the expression of the target proteins.

### 4.3. Protein Purification

To perform the ELISpot assay, human MSLN protein was extracted and purified. The MSLN gene with His tag was constructed onto the prokaryotic expression vector pET30a and was transformed into BL21 expression receptor cells. Selected bacterial colonies were inoculated into LB broth containing kanamycin and incubated at 37 °C with shaking at 220 rpm until the culture reached an OD_600_ of 0.6–0.8. Protein expression was then induced under IPTG (0.5 mM) condition at 18 °C with shaking at 180 rpm for 16–18 h. The bacteria was collected by centrifugation and lysed by sonication in an ice-water bath, and the supernatant was then collected and filtered through a 0.22 μm membrane filter. The protein was purified by a Ni column and eluted through a gradient concentration of imidazole.

### 4.4. In Vivo Immune Strategies

For immunogenicity analysis, the vaccine plasmid or vector was intramuscularly injected into the tibialis anterior muscles of both hind limbs (50 μg in each limb) of C57BL/6 mice through electroporation on days 0, 2 and 5. The mice were euthanized one week after the last immunization.

For antitumor effect analysis, 2 × 10^6^ MSLN-EGFP^+^ Panc02 cells were subcutaneously injected into the right hind flank of the C57BL/6 mice. In the first therapeutic experiment, a homologous prime-boost immunization strategy (3D + 2D) was applied to enhance immune responses. Tumor inoculation was performed on day 0, and DNA vaccines were administered intramuscularly on days 8, 10, 13, and days 22, 24. Mice were euthanized 7 days after the final immunization for sample collection and analysis. In the second therapeutic experiment, mice were challenged with tumor cells on day 0. DNA vaccines were administered intramuscularly on days 8, 10, 13. Gemcitabine (Hansoh, Lianyungang, China) was administered via intraperitoneal injections on days 8, 11, 15 and 18. Mice were euthanized 5 days after the final gemcitabine injection (i.e., 10 days after the last DNA immunization) for subsequent analysis. Mice body weights and tumor weights were measured by electric scale. The tumor inhibition rate (TIR) was calculated by the following formula: TIR (%) = [(average tumor weight of negative control group-average tumor weight of treatment group)/average tumor weight of negative control group] ×100%. The tumor size was measured every two days with a vernier caliper, and the tumor volume was calculated by the formula V = (length × width^2^)/2 (mm^3^). According to ethics guidelines, mice were euthanized if tumor volume exceed 1500 mm^3^ or if the health status of the mice was severely affected.

### 4.5. Preparation of Single-Cell Suspension

Spleens from the vaccinated mice were harvested, mechanically dissociated, and filtered. Red Blood cells were lysed by RBC Lysis buffer (BioLegend, San Diego, CA, USA). The resulting cell suspensions were washed and resuspended in R10 (RPMI-1640 (Hyclone, Cytiva, USA) containing 10% FBS) medium to obtain a single cell suspension at a concentration of 1 × 10^7^ cells/mL.

Tumor tissues were excised and placed in 6-well plates, and the tissues were thoroughly minced with sterile scissors and digested with liberase (Roche, Basel, Switzerland) and DNase I (Sigma, St. Louis, MO, USA) at 37 °C in a CO_2_ incubator for 2 h. The resulting tumor tissue suspensions were then processed following the same protocol as for splenocytes including dissociation, filtration, RBCs lysis and resuspension in R10 medium for downstream assays.

### 4.6. IFN-γ ELISpot Assays

IFN-γ ELISpot assays were performed by the ELISpot kit (BD Biosciences, Franklin Lakes, NJ, USA) according to the manufacturer’s instructions. Briefly, plates were coated with anti-mouse IFN-γ antibody overnight at 4 °C and blocked with R10 for 1 h at 37 °C before cell seeding. Splenocytes were subsequentially seeded with 1 × 10^6^ cells/well and stimulated with MSLN protein (5 μg/mL) or peptide (10 μg/mL). Cells stimulated with Concanavalin A (ConA) served as the positive control, while those cultured with medium alone were used as the negative control. The plates were incubated at 37 °C in a CO_2_ incubator for 24 h. Specific spots were then developed using biotinylated detection antibody, streptavidin-HRP, and AEC substrate. Results were quantified using an automated ELISpot reader and expressed as SFU/10^6^ cells.

### 4.7. Cell Staining and Flow Cytometry

For extracellular staining of splenocytes or tumor cells, after 2 × 10^6^ cells were suspended in 100 μL cell staining buffer (Biolegend, USA), cells were pre-incubated with anti-mouse CD16/32 (BioLegend, USA) on ice for 15 min in the dark to minimize non-specific binding. Subsequently, cells were labeled with fluorophore-conjugated antibodies and incubated on ice in the dark for 15 min.

Following extracellular staining, cells were fixed and permeabilized according to the manufacturer’s instructions using a cell fixation/permeabilization kit (eBioscience, San Diego, CA, USA) for intracellular staining. The supernatant was discarded and the remaining cells were suspended in 100 μL staining buffer. After incubation at room temperature in the dark for 15 min, 1–2 μL of fluorophore-conjugated antibody was added, followed by an additional 30 min incubation at room temperature in the dark.

After staining, cells were centrifuged at 350× *g* for 5 min, and the supernatant was discarded. The cells were then washed twice with R10 medium and finally resuspended in an appropriate volume of R10 for flow cytometric analysis.

The main antibodies used in the experiment are presented in Table A1.

### 4.8. Serum Antibody ELISA Assays

Serum was isolated from mouse blood by centrifugation (3500 rpm, 0.5 h, 4 °C) and stored at −80 °C. For ELISA, 96-well plates were coated with recombinant MSLN protein (100 ng/well) overnight at 4 °C. After washing with PBST, plates were blocked with 200 μL 3% BSA for 2 h at 37 °C. Serum samples were diluted with PBS (1:10) and 100 μL were added and incubated for 2 h at 37 °C. Subsequently, the plates were washed by PBST three times under agitation, followed by the incubation with HRP-conjugated goat anti-mouse IgG (Jackson ImmunoResearch, West Grove, PA, USA) (1:10,000) for 40 min at 37 °C. The reaction was developed with TMB substrate (Dingguo, Beijing, China) (100 μL/well) for 20 min in the dark, stopped with 2 M H_2_SO_4_, and the absorbance was measured at 450 nm using a microplate reader (Bio-Rad, Hercules, CA, USA).

### 4.9. Quantitative Real-Time PCR (qRT-PCR)

Total RNA was extracted from tumor tissues with TRIzol reagent (Invitrogen, USA), and mRNA was then reverse-transcribed into cDNA using the PrimeScript 1st Strand cDNA Synthesis kit (Takara, Tokyo, Japan). The relative expression of target gene mRNA was normalized to GAPDH and calculated using the 2^−ΔΔCt^ method.

### 4.10. Statistical Analysis

All experiments were performed with at least two independent replicates. Statistical analysis and graph generation were conducted using GraphPad Prism 8 software. Data are presented as mean ± standard error. Unpaired *t*-tests were used for comparisons between two groups, while one-way ANOVA was applied for comparisons among multiple groups. Tumor growth curves were analyzed using two-way ANOVA. A *p*-value of less than 0.05 was considered statistically significant. Except where specifically noted, all significant analyses are compared with the VR group. * *p* < 0.05; ** *p* < 0.01; *** *p* < 0.001; **** *p* < 0.0001, and ns: no significance.

## Data Availability

The datasets used and/or analysis during the current study are available from the corresponding author on reasonable request.

## Supplementary Materials

The following supporting information can be downloaded at https://www.mdpi.com/article/10.3390/ijms27042039/s1.

## Appendix A

**Table A1:** The main antibodies used in flow cytometry.

Antibodies	Source	Identifier
FITC anti-mouse CD3ε antibody	Biolegend	AB_312671
APC anti-mouse CD8a antibody	Biolegend	AB_312751
BV510 anti-mouse CD8a antibody	Biolegend	AB_2563057
BV605 anti-mouse CD8a antibody	Biolegend	AB_2562609
PE/Cyanine7 anti-mouse CD4 antibody	Biolegend	AB_312729
APC/Cyanine7 anti-mouse CD4 antibody	Biolegend	AB_312727
PE anti-mouse Ki67 antibody	Biolegend	AB_2716014
PE/Cyanine7 anti-mouse CD45 antibody	Biolegend	AB_312979
BV421 anti-mouse CD45 antibody	Biolegend	AB_2562559
APC anti-mouse CD25 antibody	Biolegend	AB_312861
PE anti-mouse FOXP3 antibody	Biolegend	AB_2936574
FITC anti-mouse CD4 antibody	Biolegend	AB_313689
PE anti-mouse CD45 antibody	Biolegend	AB_2563598
PE anti-mouse CD11b antibody	Biolegend	AB_312791
APC anti-mouse Gr-1 antibody	Biolegend	AB_313377
APC anti-mouse CD206 antibody	Biolegend	AB_10900231
FITC anti-mouse F4/80 antibody	Biolegend	AB_893502
PE/Cyanine7 anti-mouse CD86 antibody	Biolegend	AB_3106036
