# Supplementary material for: A DNA Vaccine Incorporating the MHC Class I Trafficking Domain and PADRE Epitope Enhances Antitumor Immunity in a Murine Pancreatic Cancer Model
Source: Int J Mol Sci. 2026 Feb 22;27(4):2039. doi: 10.3390/ijms27042039 (PMC12940583; doi:10.3390/ijms27042039)
Supplement: Supplementary file 1 [file ijms-27-02039-s001.zip › ijms-4123223-supplementary.pdf]

Supplemental Information

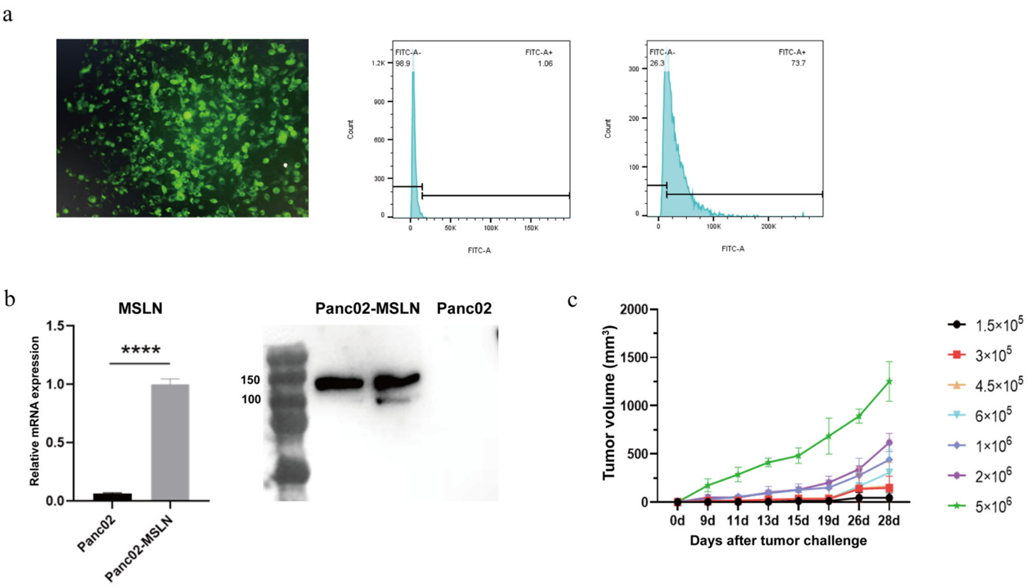

Figure S1. The establishment and validation of MSLN stable-expressed pancreatic cancer mouse model. (A) EGFP fluorescence detection signals (left) and flow cytometry analysis (right) of the MSLN-EGFP$^+$ Panc02 stable cell line. (B) MSLN mRNA expression levels (left) and Western blot analysis of protein expression (right) in the MSLN-EGFP$^+$ Panc02 stable cell line. (C) Tumorigenic dose-exploration of the MSLN-EGFP$^+$ Panc02 stable cell line.

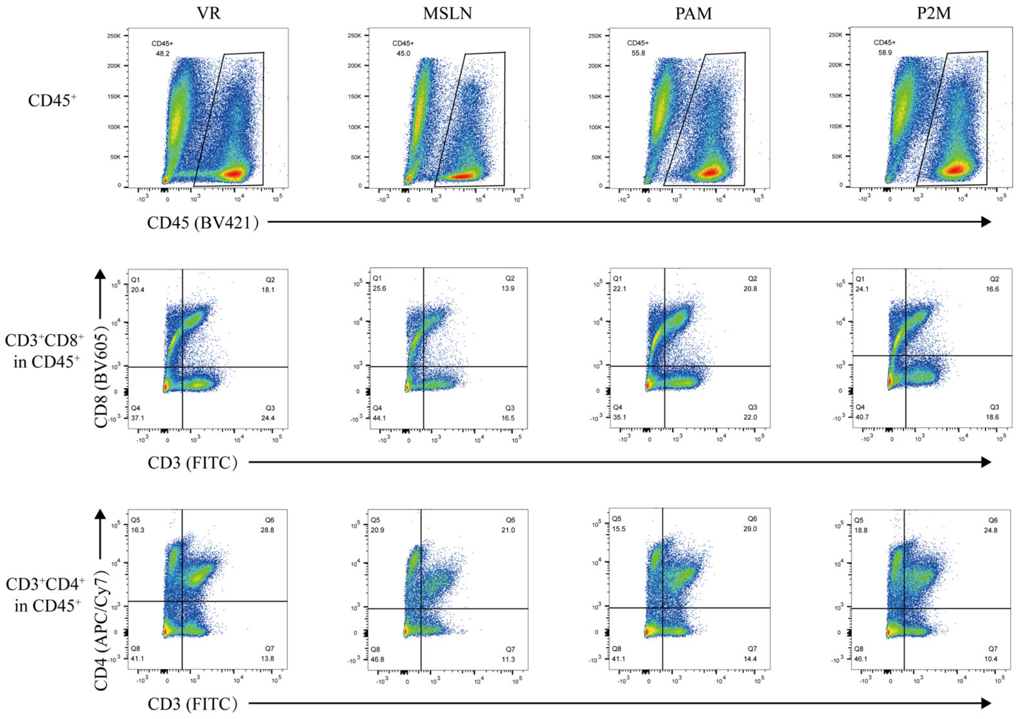

Figure S2. Representative Images of Infiltrating Immune Cells in Tumors using Flow Cytometry. These images represent the proportion of $CD45^+$, $CD8^+$ and $CD4^+$ T cells following DNA vaccine immunization.

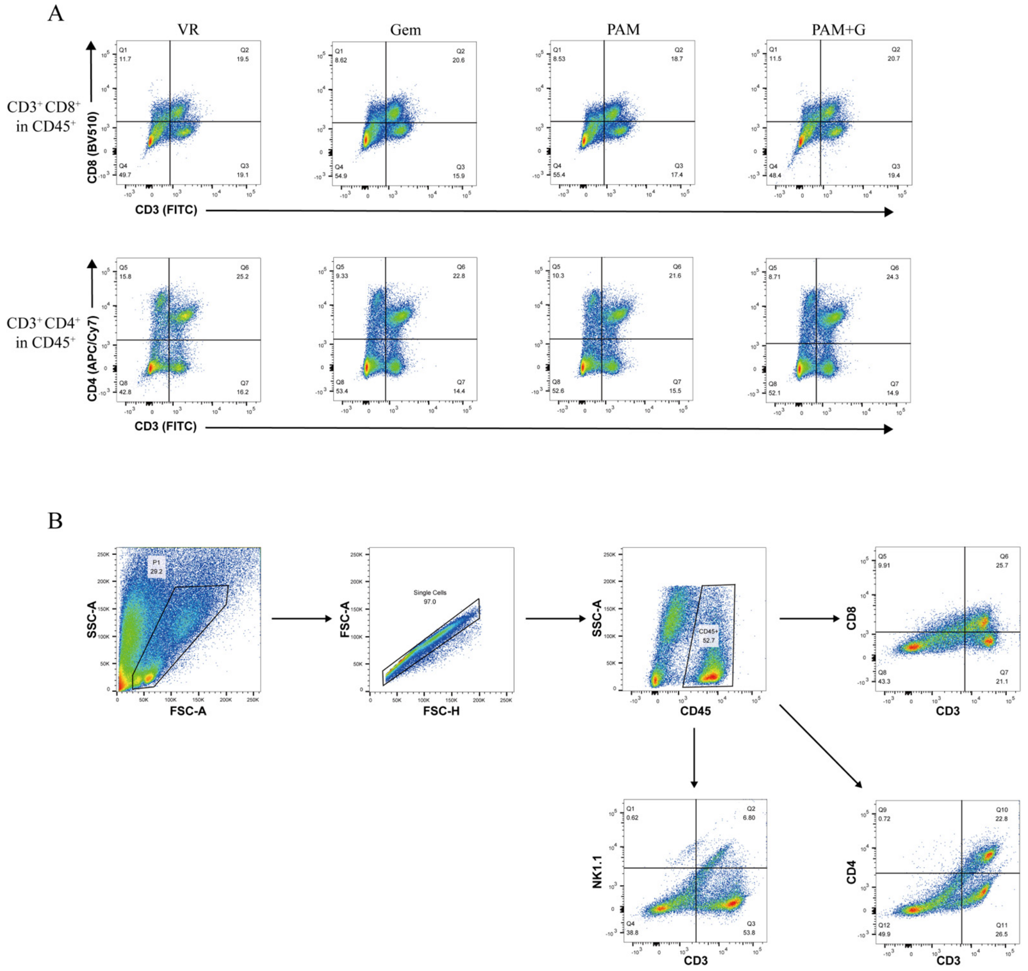

Figure S3. Flow cytometry Images of Infiltrating Immune Cells in Tumors after Gem combination therapies. (A) These images represent the CD8+ and CD4+ T cells in tumors after Gem combination therapies. (B) Representative gating strategy for flow cytometry experiments. The gating strategy of other cells in tumor is the same as the NK strategy in the figure, and different markers are shown as the ordinates in each figure.
